# Delivering transgender-specific knowledge and skills into health and allied health studies and training: a systematic review

**DOI:** 10.1007/s00787-023-02195-8

**Published:** 2023-04-28

**Authors:** L. Jecke, F. D. Zepf

**Affiliations:** 1 Department of Child and Adolescent Psychiatry, Psychosomatic Medicine and Psychotherapy, Jena University Hospital, Friedrich Schiller University Jena, Jena, Germany; 2 German Center for Mental Health (DZPG), Site Jena-Magdeburg-Halle, Jena, Germany; 3 Center for Intervention and Research on Adaptive and Maladaptive Brain Circuits Underlying Mental Health (C-I-R-C), Jena-Magdeburg-Halle, Jena, Germany

**Keywords:** Transgender, Education, Training, Health, Allied health

## Abstract

Many transgender individuals face inequities, discrimination, and sometimes even a lack of transgender-specific knowledge in health care settings. Educational curricula can address such disparities and help future health professionals to become more knowledgeable, confident, and well-prepared for addressing the needs of transgender individuals. This systematic review aims to summarize current training interventions about care of transgender individuals for health and allied health students, and to analyse the effects of the respective intervention. A total of six databases (Pubmed, MEDLINE, Scopus, Web of Science, Embase and SciSearch) were screened for original articles published between 2017 and June 2021. Search terms and eligibility criteria were pre-specified, and after a structured selection process 21 studies were included into further analysis. Extracted data contained information on general study properties, population, design, program format and outcomes of interest. A narrative synthesis was used to summarize detected results. Study quality was assessed for each individual study. A self-developed 18-item checklist combining criteria of two prior published tools was used to assess overall quality of quantitative studies. For qualitative studies a 10-item checklist by Kmet et al. [HTA Initiat, 2004] was applied. Eligible studies were designed for multiple health or allied health profession students, and varied widely regarding program format, duration, content, and assessed outcomes. Almost all (*N* = 19) interventions indicated improvements in knowledge, attitude, confidence and comfort levels or practical skills concerning care for transgender clients. Major limitations included the lack of long-term data, validated assessment tools, control groups and comparative studies. Training interventions contribute to prepare future health professionals to deliver competent and sensitive care and which may improve the prospective experienced health care reality of transgender individuals. However, currently there is no common consensus about best practice of education. Additionally, little is known about whether detected effects of training interventions translate into noticeable improvements for transgender clients. Further studies are warranted to assess the direct impact of specific interventions in the light of the respective target populations.

**Supplementary Information:**

The online version contains supplementary material available at 10.1007/s00787-023-02195-8.

## Introduction

According to article 12 of the International Covenant on Economic, Social and Cultural Rights of the UN) every human being has the right to the highest attainable standard of physical and mental health [2]. Essential and interlinked core components to achieve this objective are the following: Availability (which refers to a sufficient quantity of facilities, goods and services), accessibility (including non-discrimination policies, physical accessibility, affordability and access to information), acceptability (implies provision of cultural appropriate and respectful care) and quality [3].

Despite these demands for equality increasing research activities on the health of trans or transgender individuals identified major health disparities when compared to the general population, which will be further specified below. Transgender individuals are people who have a gender identity and/ or expression that differs from their sex assessed at birth, whereas people whose gender identity and gender expression match the sex assessed at birth are referred to as cisgender by some people [4]. However, there is still an ongoing controversy about the use of the term cisgender because some people who would fall under this particular term reject to be categorized as “cis”, in particular because this term originally stems from chemical science and was not developed to describe human identity perception in the first place.

The more inclusive umbrella term trans describes a diverse group of people who identify as transgender, transsexual cross-dressing, androgyne, polygender, genderqueer, agender, gender variant, gender non-conforming or with any other gender identity and/or expression which does not meet the societal and cultural expectations placed on gender identity [5].

There are no exact data about the size of the transgender population, in particular because many assessments rely on ICD codes [6], or only capture individuals who seek gender affirming care at specialized clinics [7], and which may result in rather underestimated figures. Studies who interviewed general population samples about their gender identity created higher appraised values, ranging from 0.5 to 1.3% for birth-assessed males, and from 0.4 to 1.2% for birth-assessed females [7].

The results of two surveys with large numbers of transgender respondents conducted in the U.S.A. and EU [8, 9] demonstrated high levels of experienced discrimination, violence, and harassment in different areas of life such as work, education, and health care. In addition, they experience poverty more frequently or have a lower household income when compared to the general population or other Lesbian, Gay or Bisexual (LGB) groups.

When asking members of the trans community in the EU if the government effectively combats prejudice and intolerance against Lesbian, Gay, Bisexual, Trans or Intersex (LGBTI) people definitely or probably, only 24% believe that this is the case [10]. But 94% stated they could live more comfortably if national authorities promoted the rights of trans people [8]. The call on States for protecting LGBTI individuals from discrimination and violence in all contexts and life situations is supported by United Nations entities. They demand an urgent response to current circumstances from governments, parliaments, judiciaries, and national human rights institutions and also emphasise that health providers play important roles [11].

Regarding health care many transgender individuals report negative experiences with health providers like verbal harassment because of their gender identity, non-affirmation by using incorrect pronouns and sometimes professionals did not seem to want to help or even refused to provide treatments [8, 9]. Another important problem is the denial of gender-affirming care by insurance providers [9]. These circumstances and the fear of being mistreated can lead to avoidance of health care services and other negative physical and mental health effects [5, 12]. Studies have shown that transgender people are considered to be burdened with higher rates of mental health concerns when compared to the general population, including depression, anxiety, self-harm and suicide [13–15]. These increased negative mental health outcomes were also found among transgender youth [16].

Such differences could at least partially be explained within the theory of minority stress implying that discrimination, violence, stigma, and prejudice “create a hostile and stressful social environment” [17] that is the cause of a higher prevalence of mental health problems [18]. Minority stress constructs (distal stress, internalized transphobia—we would prefer the term cissexism-, expectations of rejection, and concealment) were reported to be significantly associated with increased depression, suicidal ideation, and suicide attempts [19].

Accordingly, there is an explicit necessity for gender affirmative health care which considers that every person has individual needs. It can include any single or combination of social, psychological, behavioural or medical (including hormonal treatment or surgery, although so far there is only limited evidence available) interventions designed to support and affirm an individual’s gender identity [20]. Several studies documented that mental wellbeing is enhanced by using ones chosen name or having hormonal or surgical interventions, as was shown by reduced rates of suicidal ideation and suicide attempt, fewer depressive symptoms, and increased life satisfaction [21–24]. After initiation of puberty blockers and gender-affirming hormones significantly lower odds of depression and suicidality were detected among transgender youth [24]. However, the overall certainty about such interventions is very low. Moreover, many providers evidently lack in appropriate knowledge about general health needs (for example specific risk factors for potentially co-varying conditions) as well as about more specific medical aspects (for example so-called gender affirming care) and life situation of transgender adults and youth and skills, which is important to perform competent and sensitive care [25–29].

Some transgender youth report on positive experiences in gender-affirming speciality clinics where they felt safe to discuss problems and were called by their chosen name all the time [30].

On this account, education and practice are essential to address such gaps, in particular because the paucity of training and shortage of exposure to transgender clients are regarded as main barriers [31]. A study with providers who care for transgender youth reported though 62% felt comfortable with providing medical therapy, and only 47% felt confident in their abilities. Most frequently named barriers that interfered with provision of transgender-related are were a lack of training and exposure, as well as a lack of mental health sevrice providers to collaborate with [31].

Therefore, multiple educational interventions were created aiming to educate providers and professionals about needs of transgender clients and to reduce current health disparities.

Donisi et al. [32] piloted a multimodal training project on LGBTQI health, with one module specifically dedicated to transgender and intersex health needs. Participants were healthcare professionals from diverse medical fields and 6 European Member states. Effectiveness was evaluated comparing pre- and post-survey answers. Knowledge and attitude scores were increased after the training intervention, and which indicated an effective promotion of inclusive healthcare practice. Further research confirmed the promising results of educational interventions about needs of sexual and gender minorities on health providers and students’ knowledge, attitudes, or skills [33–38].

The aim of this systematic review was to summarize these current training interventions about care of transgender individuals’ content in curricula of health or allied health profession students. There are current literature reviews about education on transgender health care which often included only one health profession, for example like medical students and residents [39] or pharmacists [40]. But because health care is a multidisciplinary task we incorporated all students of health professions (e.g. nursing, medicine or Physician Assistant) or allied health professions [41] in this review in order to give a broader overview. Another important feature is to highlight education exclusively on transgender care since previously published reviews often studied interventions about LGBTQI health in general [42–45] or cultural competency [46] and did not focus on a special population.

The two main questions we aimed to address were the following:

1. Which kind of interventions do currently exist to educate about care for transgender clients?
2. Which influence do training interventions of future health professionals have on care of transgender people?

## Methods

We conducted an electronic literature search in six major databases including Pubmed, MEDLINE (Ovid), Scopus, Web of Science, Embase and SciSearch (STN International) between 22 Mai 2021 and 17 June 2021. The Preferred Reporting Items for Systematic reviews and Meta-Analysis (PRISMA) 2020 statement [47] were used as guidance to provide a transparent research approach and synthesis of found results.

### Search strategy

Studies were included if they presented any kind of training intervention (e.g., lecture, patient simulation, panel discussions) to educate students about transgender care, and if there was an evaluation of intervention outcomes.

To decide whether articles were eligible the PICO approach defined relevant study populations, educational interventions, comparators, and outcomes (see Table 1).

**Table 1:** Given are relevant elements for study inclusion using the PICO approach

Population	Students of health or allied health professions
Intervention	All kinds of training with content about care of transgender clients
Comparator (if available)	Standard curriculum or no training
Outcome	Changes in attitude, knowledge, skills competence, awareness, or similar objects in terms of care of transgender people

Further inclusion criteria were a publication date between 2017 and 15. June 2021 and English or German as publication language. Exclusion criteria involved the following: (1) reports already published in form of a review, commentary, conference abstracts or any kind of grey literature, (2) studies which were already part of a former systematic in order to avoid duplication [39, 40, 42–46]. (3) Interventions which did not exclusively focus on needs of transgender individuals were also excluded.

Search strategies of previous evidence reviews about hormone use in children and adolescents with gender dysphoria [48, 49] were studied to capture all synonyms and words related to the term transgender and to create a suitable search query.

The final search terms consisted of the following: ((Gender* and (dysphori* or incongruen or identit* or disorder* or minorit*)) or (transgend* or transsex* or transex* or transfem* or transwom* or transma* or transmen* or transperson* or transpeopl*)) and (train* or teach* or curricul*) and student* and health*

There were minor differences concerning the filters or the use of truncation in each database to narrow down search results. The complete search strategy for each database can be found in Supplementary 2b.

Hereinafter, for reasons of simplification and readability, we will continue with the term transgender for someone whose gender identity and/ or expression does not align with their sex assessed at birth (which was the most frequently used approach across the studies found in our literature search). We are aware that the term may not correctly refer people who identify as trans or outside of the gender binary.

The electronic literature search identified 4389 records across all databases. Results were imported into Endnote (Version X9.3.3), and duplicates were removed. Remaining articles were screened by title and abstract. The full version of potentially eligible records was obtained and studied to decide whether inclusion criteria with regard to the PICO approach were met, and if the report was already mentioned in a previous review. After this structured selection process performed by the first author, a total of 21 studies were incorporated for data analysis and synthesis.

**Fig. 1:**
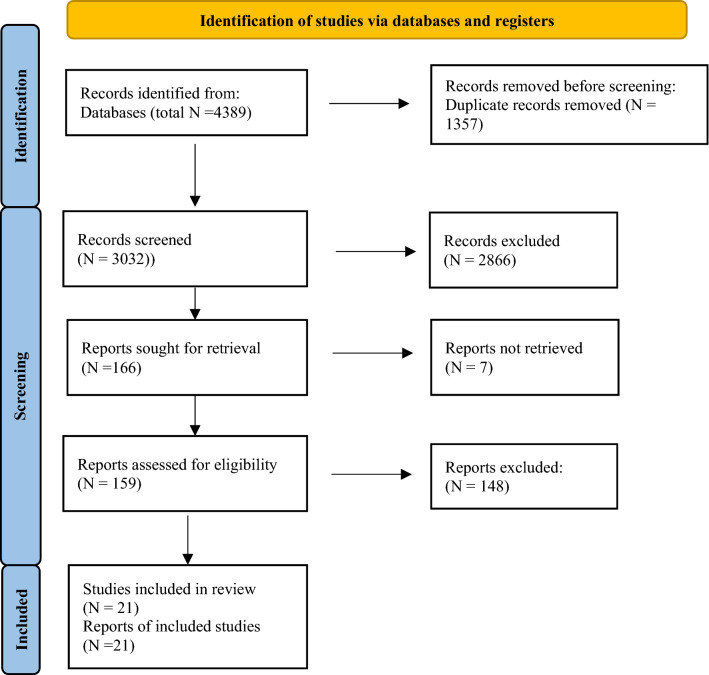
PRISMA Flow Chart of study selection (from*:* Page MJ, McKenzie JE, Bossuyt PM, Boutron I, Hoffmann TC, Mulrow CD, et al. The PRISMA 2020 statement: an updated guideline for reporting systematic reviews. BMJ 2021; 372:n71. https://doi.org/10.1136/bmj.n71)

The flow diagram in Fig. 1 illustrates the process of study selection. A more detailed presentation can be found in Supplementary 2a.

We sought information on the following: General properties (title, authors, publication year, study location), study population (number of participants, profession, voluntariness of participation), study design (data collection method, outcomes of interest) and program format (teaching method, content, time frame).

Given the heterogeneity in study population, design, and outcome measures across included studies a quantitative synthesis in the form of a meta-analysis was not appropriate. Therefore, we followed the Guidance on the Conduct of Narrative Synthesis in Systematic Reviews guideline [50] to ensure transparency using alternative synthesis methods. We analysed general study characteristics, explored the content delivered and synthesised data regarding the teaching methods and different outcome parameters. In addition, we established synthesis groups for Interprofessional education (IPE) activities and approaches of longitudinal education.

### Quality assessment

An 18-item assessment tool was used to evaluate overall quality of each included study. The authors developed the tool based on two prior published quality assessment checklists [1, 51] within an explorative approach. Relevant questions were applied to 20 studies with a quantitative research design and could be scored with “yes”, “partial”, “no” or “not applicable”. The quality assessment tool and a tabulation of all individual study scores can be found in Supplementary 4a–c. An item was answered with “yes” if the subject was fully addressed and described or the individual study met all requirements of the question. Accordingly, a “no” was given if these criteria were not met or the report contained no information to answer the item. When some criteria were fulfilled but not in a sufficient way the item was scored with “partial”. “Not applicable” meant that the question was not appropriate to answer because of the study design.

Our critical appraisal aimed to identify weak points to estimate the risk of bias which translates in a poor-quality rating. In principle, one expects a higher study quality the more items can be scored with “yes”.

The quality of the only qualitative study in this review by Montes-Galdeano et al. [52] was assessed using a 10-item checklist by Kmet et al. [1]. Each question can be scored with a maximum of 2 points which results in a total possible sum of 20 points for the highest quality.

None of the studies was excluded due to low quality ratings if all inclusion criteria were met.

## Results

The 21 eligible reports are represented in Supplementary 3 categorized by intervention characteristics (learning modalities, content, time), number and profession of study population, voluntariness of participation, data collection methods and outcome measures.

### Study characteristics

General study characteristics turned out to be highly variable. Almost all studies (*N* = 18) were conducted in the USA, followed by Spain (*N* = 2) and Australia (*N* = 1). The student population represented a big range of health-allied health professions. Most training interventions were designed for medical students (*N* = 9) and nursing students (*N* = 9), and some studies focused on pharmacy students (*N* = 3) or social work students (*N* = 3). Further included professions were physician assistant, occupational therapy, physical therapy, speech language pathologist, public health, rehabilitation therapy and few studies (*N* = 2) added experienced learners like residents or general practitioners. Three interventions were created as an IPE [53–55], hence given to more than one profession. Number of participants varied from 11 to 494 students.

Teaching methods included didactic lectures and seminars in a classroom setting or online format (*N* = 9) as well as standardised patient simulations (*N* = 7). The simulation patient is portrayed by trained staff, a person identifying as transgender or a manikin. One study designed a dyadic simulation scenario around a parent and transgender child [56]. Three interventions had a hybrid approach combining simulation with lecture, panel presentation or didactic presentation and role plays. Five studies integrated multimodal concepts containing patient panels, group work and video discussion. The opportunity for students to face members of the transgender community during panel discussions or as standardized patients was provided by nine different interventions.

Most studies applied quantitative data collection methods of different outcome parameters (*N* = 16) with self-assessment tools (*N* = 15) or external assessment by facilitators (*N* = 1). Four studies used a mixed methods approach combining questionnaires and the option of free-text and one report had a qualitative research design with focus group interviews. A pre/post-survey concept was implemented in 16 interventions, whereas four studies only used postinterventional surveys. Long-term data 1 year after completing the intervention were exclusively presented by one study [57].

Almost all reports had a non-randomised intervention design without control (*N* = 18). Three studies established a control group [58–60], and one of them additionally performed a randomization of students to two different intervention groups [60].

Teaching time most frequently was 2 h and below (*N* = 8), but seven training interventions took longer than 2 h. Six projects did not provide exact time data or only mentioned the duration of some intervention components. The intervention usually took place on one day (*N* = 16), yet in some studies (*N* = 5) students participated for more than 1 day up to 3 semesters with several educational activities.

Teaching on care of transgender individuals was an integrated part of the standard curriculum (*N* = 14) either for all students or within a special course or were offered extracurricularly (*N* = 7). Four interventions were embedded within a longitudinal curriculum on various transgender related or LGBTQ related health topics. Students’ participation was equally often voluntarily (*N* = 9) or as required (*N* = 9). Three studies did not provide information about attendance status.

### Content of educational interventions

Overall content of training interventions was much diverse, but several key topics were identified. Almost all studies (*N* = 19) informed their students about gender-affirmative care in general or about some aspects like social affirmation (including chosen name and pronouns, *N* = 12), medical affirmation (including hormonal and surgical practices, *N* = 11) or legal affirmation (including updating name on legal documents, national policies, *N* = 7). Further common topics were health inequities and barriers in health care access faced by transgender individuals (*N* = 12), and best ways of respectful, culturally sensitive provider–patient communication (*N* = 12), particularly regarding medical history taking or physical examination. Moreover, several studies involved the following content: Terminology like defining “gender identity” and the term “transgender” (*N* = 14), discrimination and stigmatization (*N* = 11), mental health concerns (*N* = 8), diagnostic criteria of gender dysphoria in DSM V (*N* = 7), preventive health especially cancer screenings (*N* = 6) and resources and referrals for supportive care (*N* = 7). Five studies provided knowledge about special challenges when working with adolescents or children identifying as transgender, and only three projects integrated content about nonbinary individuals explicitly (Table 2).

**Table 2:** Data extraction of individual studies

References	Intervention	Participants	Outcome assessment	*N* for assessment	Results
Hart [64] (2021) USA	Teaching method Lecture Content Terminology, unique barriers to health, legal topics, community resources, transgender-specific history and physical exam skills, and published guidelines for medical and surgical management of gender dysphoria Duration 2 h Requirement status Voluntarily	169 Physician assistant students	15 self-assessment questions about knowledge (2 items), skills (2 items), and attitudes (2 items) regarding transgender topics on a 7-point Likert scale (0 = very low; 6 = very high) and 9 opinion statements on a 5-point Likert scale (1 = strongly agree; 5 = strongly disagree)	98	Significantly improved: postlecture knowledge and skills scores (*p* < 0.0001) No significant improvements in attitudes but some significant positive changes in opinion statements
Stumbar [68] (2018) USA	Teaching method Workshop with standardised patient scenario + lecture Content Components of an inclusive sexual history based on the 5Ps model, transgender health topics: mental health concerns, substance use, personal safety, hate crimes, sexual history including pregnancy goals, gender-affirmation process, and gender identity and sexual orientation terminology Duration 2.5 h Requirement status Mandatory	113 second year medical students	Survey consisting of multiple choice and Likert-type questions (5-Point scale) assessing changes in knowledge and self-reported attitudes and skills related to the sexual history and to transgender patient care, as well as satisfaction with the session itself	73 answers to knowledge question 110 answers to all other questions	Significantly improved: students’ knowledge about 5Ps model, students who felt effectively prepared to provide medical care and felt comfortable gathering a sexual history from a transgender patient > 90% of the students believed that they were better equipped in knowledge and skills to effectively provide medical care and a majority thought the standardised patient case was realistic and believable and debriefing session helpful
Ostroff [58] (2018) USA	Teaching method Lecture Content 70% of the lecture focused on cultural sensitivity and 30% on pharmacotherapy Duration 2 h Requirement status Mandatory	Pharmacy students (intervention group: 72 P3 students, control group: 69 P4 students)	11 knowledge-based yes/no questions and rating of confidence with their answer on a 5-point scale 2 additional questions with a 5-point scale related to the participant's comfort level with providing care or prescription counselling to a transgender patient	71 (IG) 38 (CG)	P3 class had a significantly higher mean knowledge score than the P4 class (72.5% vs. 63.4%, *p* < 0.01) and significantly higher confidence score than the P4 class (76.8% vs. 60.6%, *p* < 0.01) No difference in reported mean comfort level for providing care (P3 = 3.33 vs. P4 = 3.35, *p* = 0.955) or medication counselling (P3 = 3.41 vs. P4 = 3.11, *p* = 0.295)
Sherman [63] (2020) USA	Teaching method Different forms of seminars and lectures integrated in 5 core courses Content Health inequities, gender-affirming language and best practices, preventive health, mental health, gender-affirming interventions, and prenatal care Duration Longitudinal over 3 semesters Requirement status Mandatory	160 nursing students enrolled in an accelerated BSN program	Adapted version of Kelley et al. measure of knowledge and attitudes regarding LGBT health (8-items, 5-point Likert scale; 1 = strongly disagree, 5 = strongly agree)	T1 *n* = 80 T2 *n* = 43 T3 *n* = 31	Significant increase: level of importance nursing students assigned to knowing their patient’s gender identity between T1 and T3 as between T2 and T3, participants’ confidence in their skills to provide respectful and effective care between T1 and T3, greater awareness of resources for information and referral specific to Transgender patients between T1 and T3 but scores for this item remained below the threshold for being considered gender sensitive No other changes were significant
Turban [71] (2018) USA	Teaching method 2 lectures Content Terminology, diagnostic criteria for Gender dysphoria (GD), psychiatric co-morbidities, medical interventions, resources to support transgender youth Duration No time data available Requirement status Mandatory	407 first to fourth year medical students	7 knowledge questions (one with multiple components) and a single dichotomous choice item asked whether hormonal therapy for transgender paediatric patients was ethical or unethical	162	Students performed well on most knowledge questions and less well on inquiries about GnRHa (65%) and spironolactone (38%) and time requirement to meet criteria for GD diagnosis (36%) No significant differences in performance knowledge questions by training year, except for questions on the time requirement for a GD diagnosis and benefit of spironolactone Majority of students (86%, *n* = 139) felt that it is ethical to administer hormonal interventions
Thompson [69] (2019) USA	Teaching method Multimodal Content Cultural competency related to gender identity and sexual orientation, health disparities, history, development and function of the gender dysphoria diagnosis, challenges for TGNC individuals, Minority Stress Theory, mental health concerns, legal and social gender-affirming strategies, medical management of the adolescent patient, feminising and masculinising treatment options, preventive healthcare screenings and fertility counselling Duration 6 h 35 min Requirement status Mandatory	127 s year medical students	Adapted version of the Sexual Orientation Provider Competency Scale consists of 26 items and three subscales (skills, attitudes, and knowledge), with 5-point Likert response options (1 = strongly agree, 5 = strongly disagree) Two additional questions were assessed: ‘I have an interest in issues that transgender and non-binary people face, outside of the healthcare setting’ and ‘I have used or role-played the use of pronouns and chosen name in clinical settings’	*n* = 100 for preassessment *n* = 81 for postassessment *n* = 77 completed both	Significantly improved: overall score, knowledge and skills subscale, students’ experience using/role-playing pronouns in a clinical setting No statistically significant difference: attitudes subscale, interest in TGNC issues outside of the healthcare setting
Congdon [61] (2021) USA	Teaching method Seminar (5 different occasions) Content Terminology, diagnosis and prevalence of gender dysphoria per the DSM-5, nonhormonal treatment (e.g., surgery, mental health treatment for common comorbidities), gender-affirming pharmacotherapy, and other primary care–related considerations (e.g., organ-specific cancer screenings, HIV preexposure prophylaxis, renal dosing of medications, cardiovascular disease risk, bone health, family planning) Duration 30–45 min Requirement status Voluntarily	100 participants of different medical departments (7 medical students, 5 pharmacy students, residents, doctors, pharmacists, pharmacy technicians and more)	Trans* Health Education Evaluation Scale (THEES) was developed to assess participants’ self-perceived proficiency and confidence in providing care to gender-diverse patients Two additional questions on posteducation survey to assess participants` confidence level and direct clinical applicability: 1. This discussion has increased my confidence level for providing care to transgender individuals 2. I learned something from this discussion that I can directly apply to my clinical setting Each item was rated on a 5-point scale (1 = strongly disagree to 5 = strongly agree)	100	Significantly improved: THEES total scores, each individual scale item (*p* < 0.001) Majority of participants felt that the seminar was directly applicable to their clinical setting by 90% responding with agree or strongly agree. In the same manner, 84% of participants felt an increase in confidence
McCave [53] (2019) USA	Teaching method Standardised patient scenario + panel presentation of local community Content Interprofessional core competencies and standards for affirmative practice for transgender patients Duration ~ 2,5 h Requirement status Mandatory for 95% of students	494 graduate health care learners in medicine, nursing, occupational therapy, physical therapy, physician assistant, social work, and health care administration programs over 2 years	Surveys collected satisfaction data with each component (rated on a 5-point scale) and data on ability to practice the four IPEC core competencies (adapted version of Alan Dow’s 2012 IPEC Competency Survey with a 5-point Likert scale)	278	Averaging of 2 years of data yielded student responses of strongly agree or agree at 90% or higher for all IPEC core competencies [preparation to maintain values, ethics, and affirming practice (93%), teamwork (90%), effective communication (90%), and collaboration with a variety of health care professions (91%)] 93% identified the addition of transgender individuals sharing their personal experiences as useful or very useful. The discharge planning meeting with the standardized patient got the second highest rating (85%)
Montes-Galdeano [52] (2021) Spain	Teaching method Standardised patient scenario Content Nursing assessment with a transgender patient, terminology, discriminatory attitudes against transgender individuals Duration Time data not available for the simulation scenario, 40–60 min for focus group interviews Requirement status Voluntarily	135 first year nursing students	Qualitative data gathering through 12 focus groups (FG) interviews with 9–11 students in each group, each focus group was led by two researchers, one specialist in lead group dynamics and the other who assisted him and took the field notes. A semi-structured interview guide was used	124	Self-awareness about LGBTQ + individuals’ health care needs was increased and importance of their approaches to achieve a positive impact on transgender patients’ predisposition to seek help in nurses, participants had positive and respectful attitudes towards LGBTQ + individuals but negative attitudes of the nurse during the handover were not criticized by the majority, including LGBTQ + contents into their curricula is essential, lack of experience when communicating with trans patients
Najor [57] (2020) USA	Teaching method Lecture Content Explanation of the spectrum of identities associated with gender expression and sexual orientation, a broad overview of LGBT + health disparities, and description of a patient scenario to demonstrate how subtle aggressions by medical staff may lead to less health care utilization and poorer treatment outcomes Duration 1 h Requirement status Mandatory	100 first year medical students	21-question survey focused on comfort level with treating transgender patients and their personal beliefs and experiences with transgender people. Knowledge questions could be answered in yes/no or true/false format, whereas attitude questions could be answered with a four-level scale for comfort or agreement	*n* = 86 prelecture survey *n* = 70 postlecture survey *n* = 44 1-year follow-up survey	Postsurvey: statistically significant increase of students who were comfortable caring for transgender people and with the described patient scenario, proportion of students who were aware that transgender people have unique health risks and health needs significantly decreased 1-year follow-up: significant increase in number of students who were comfortable with the described patient scenario (compared to prelecture) (*p* < 0.0001). The proportion of students who were aware that insurance companies are increasing coverage for transgender health care changed significantly
Muckler [66] (2018) USA	Teaching method Standardised patient scenario Content Brief presentation of trans information and health disparities, culturally competent communication during anaesthesia preoperative interview with a transgender simulation patient Duration No exact time data available, 40 min for debriefing session Requirement status Voluntarily	30 first, second-, and third-year students in a 3-year Doctor of Nursing Practice nurse anaesthesia program	6 yes/no questions that assessed students' perceived comfort and competence providing care to trans patients. The survey was adopted from the LGBT Health Issues Questionnaire with an opportunity for students to comment on their experiences	28	Ongoing feelings of incompetence related to the health care needs of trans patients Simulation was helpful, effective, and increased their ease of caring for trans patients Desire for further trans content and simulation training Most students did not know how to address the patient’s requests to have the name on her armband changed and to have both testes removed during the emergency surgery
Maruca [67] (2018) USA	Teaching method Standardised patient scenario Content Therapeutic communication in a culturally sensitive manner with a transgender patient, identify signs and symptoms and managing anxiety Duration No data available Requirement status Voluntarily	159 undergraduate, prelicensure BSN nursing students	Gay Affirmative Practice (GAP) survey, a 30- item self-report survey using a 5-point scale, examines attitudes and beliefs to evaluate affirmative practice that models culturally competent care to gay, lesbian, and bisexual populations. Questions 1 to 15 measure a practitioner’s beliefs and attitudes about treatment; questions 16 to 30 measure a practitioner’s behaviours in the clinical settings. Responses are “strongly agree” (5) to “strongly disagree” (1) for the first 15 questions and “always” (5) to “never” (1) for the second group of questions	48	Significantly improved: overall GAP score, median score of behaviours subscale Minimal change in median scores for beliefs/attitude but not statistically significant
Arora [65] (2019) Australia	Teaching method Seminar on three different occasions Content Appropriate transgender terminology; exploring the biological basis of gender identity and diversity; the lived experience of a transgender person and their relationship with healthcare providers; supportive care for children and families; adolescent puberty blockade; adult transition care; fertility options; hormonal monitoring and surgery Duration 1 h Requirement status No data available	Third year medical students, general practitioners, internal medicine physicians, no concrete data about number of participants	Adapted and extended survey from tools of Safer and Pearce 2013; Thomas and Safer 2015. Participants rated their comfort to facilitate care for an adult: (1) assigned female at birth requesting masculinising therapy; (2) assigned male at birth requesting feminising therapy; (3) assigned male at birth and requesting care for low testosterone levels; or (4) assigned female at birth and requesting care for low oestrogen levels and (5) their predicted comfort to support a gender-questioning child or (6) an adolescent presenting with a gender different from their sex assigned at birth (7) Additionally, 3 knowledge questions about cancer screening and 3 opinion questions were asked. Responses were collected on a 5-point Likert scale and true–false responses	*n* = 188 presurvey *n* = 163 postsurvey	In presurvey a high percentage of respondents ‘disagreed or strongly disagreed’ that they felt sufficiently knowledgeable to assist a person with therapy to transition. They showed significantly less discomfort in providing hormone therapy for hypogonadal cis-gender individuals Postsurvey: significant increase in comfort with scenarios to provide support for a gender incongruent adult, adolescent or child, number of students who agreed that hormonal and surgical treatment should be offered to the transgender community as well as knowledge about cancer screenings in transgender individuals
Klotzbaugh [62] (2020) USA	Teaching method Seminar Content Medical knowledge and health disparities, local and national policies, appropriate terminology, difference between sexual orientation and gender identity, importance of a gender minority-specific health module, initiation and appropriate management of gender affirmation hormone therapy Duration 1.5 h Requirement status No data available	11 advanced practice nursing students	11 items survey (adapted from a questionnaire by Braun, Garcia-Grossman, Quiñones-Rivera, and Deutsch (2017)). Higher scores indicated greater knowledge in medical considerations, health disparities or health policies Transphobia scale, which is a 9-item scale developed by Nagoshi et al. (2008) was used to measure prejudice against gender minorities. Items were scored on a scale of 1 (disagree) to 3 (agree), with 2 indicating neutrality	11	Significant difference: mean for each knowledge domain, mean scores of transphobia scale
Pathoulas [72] (2021) USA	Teaching method Lecture Content Gender affirming hormone therapy (GAHT), informed consent model of care, medical management of hormone therapy, terminology, diagnosis, health inequities. The interactive portion consisted of actors role-playing an office visit in which a physician initiated GAHT Duration 1 h Requirement status Voluntarily	54 Second-year medical students	Survey with 6 questions that addressed self-perceived preparedness and comfort with learning objectives using a 5-point Likert scale	54	Significantly improved: familiarity with GAHT dosing, (*p* < 0.001), common medications used in GAHT (*p* < 0.001), the use of informed consent in clinic to initiate GAHT (*p* < 0.001), their confidence in finding resources to provide GAHT (*p* < 0.001), providing GAHT in the primary care setting (*p* < 0.001) and the difference between a legal and chosen name (*p* < 0.017)
Ozkara San [59] (2020) USA	Teaching method Standardised patient scenario Content SP simulation activity with a transwoman with breast cancer experiencing an oncological emergency, PowerPoint about cancer care for transgender patients, and cultural assessment examples Duration 3 h Requirement status Voluntarily	Senior level ABSN nursing students, experimental group (*n* = 16, participating in the simulation) and control group (*n* = 52, did not participate)	83‐item TSET uses a 10‐point rating scale (1 = not confident to 10 = totally confident), measures three dimensions: Cognitive (25 items), Practical (28 items), and Affective (30 items) Simulation Effectiveness Tool—Modified (SET‐M) was adapted to evaluate if the simulation meets with students' learning need, 19 items on a 3‐point scale to assess prebriefing, simulation and debriefing 4 additional questions using a 10‐point rating scale focusing on the impact of the simulation on students' knowledge development; cultural assessment and interview skills; culturally sensitive attitudes, values, and beliefs; and overall confidence	EG = 16 CG = 41	Experimental group posttest survey: most change on the Cognitive subscale, followed by the Practical and Affective subscales, changes were statistically significant Control group posttest survey: results were statistically significant and the most change occurred on the Practical subscale, followed by Cognitive and Affective subscales The mean Cognitive, Affective, and Overall TSET scores among the CG and the EG were statistically different. No overall difference among the group means for the practical subscale
Pechak [54] (2018) USA	Teaching method Multimodal Content Introduction in IPE and overview of transgender health, discussion about the case of a transwoman with stroke and the barriers and challenges she faced while transitioning (including health care access, employment, mental health, discrimination, gender identification on legal and healthcare documents) Duration 3 h Requirement status Mostly mandatory	108 students (22 OT, 11 Pharm, 35 PT, 15 RC, 20 SLP, 5 SW)	Survey was a combination of RIPLS (readiness for interprofessional learning scale) and IPAS (interprofessional attitude scale), consisted of 37 items rated on a 5-point Likert scale (1 = strongly agree, 5 = strongly disagree) 2 additional items asked in the postsurvey about comfort when interacting with transgender individuals and comfort when providing services to transgender patients, the items were rated on a 5-point scale from 1 (lowest) to 5 (highest)	101 completed both surveys 105 completed an evaluation of the IPE event including rating the effectiveness and their comfort levels with transgender individuals	Significant changes in both scales IPAS and RIPLS Scores between two extra items were significantly different indicating that students rated their comfort levels higher for providing services than personal interactions
Allison [55] (2019) USA	Teaching method Multimodal Content Definitions and terminology, affirming care practices, four components of identity (body, mind, appearance, and attraction), transitioning process, health disparities and unique health needs, presentation highlighted state-specific results of the U.S. Trans Survey Duration 2 h Requirement status No data available	58 health profession or nursing students	Survey examined knowledge of terminology, health care practice, and attitudes toward trans/Nonbinary individuals and gender-affirming care. 10 knowledge items with 5 fill-in-the blank questions, 3 true or false questions, and 2 multiple choice questions The Transgender Attitudes and Beliefs Scale (TABS) assessing interpersonal comfort, sex/gender beliefs, and human values elicited the students’ attitudes. It consisted of 29 questions a 5-point Likert scale 3 additional questions to assess attitudes toward trans/Nonbinary patients, their responsibility as health professionals to serve those clients, and their concerns about cisgender patients knowing that they serve trans/Nonbinary patients	*n* = 56 (completed both the pre- and post knowledge survey) *n* = 51 (completed both the pre- and post-interpersonal comfort attitude subscales) *n* = 50 (completed both the remaining pre- and post-attitudes subscales)	Significantly improved: knowledge, interpersonal comfort and sex and gender beliefs on subscales No differences on the human value subscale or on health care professional questions
Berenson [70] (2020) USA	Teaching method Multimodal Content Unique health issues and disparities, description of medical transition and hormone therapy for transgender patients, pronouns, competent and poor ways of communication between a provider and a transgender patient, culturally competent practices in taking an initial history from a patient Duration 2.5 h Requirement status Mandatory	178 second year medical students	Preworkshop survey to assess their confidence in addressing each of the three learning objectives on a 5-point scale (0 = no confidence, 4 = complete confidence) and their experience with homophobic and transphobic comments on campus, postworkshop survey again assessed each learner’s confidence in addressing the three learning objectives and asked to rate the quality of the workshop components on a 5-point scale (1 = poor, 5 = excellent)	123 matched pairs	Significantly improved: mean rating of confidence levels for all three learning objectives (describe the unique health issues and disparities experienced by transgender individuals, describe medical transitioning and hormonal therapies, describe best practices for promoting culturally competent and affirming care) Overall Workshop Evaluation: mean ratings were highest for the patient panel at 4.5, compared to 3.9 for the large-group didactic lecture, and 3.8 for the small-group video session
Tyler [56] (2020) USA	Teaching method Standardised patient scenario Content Presentation about definitions, culture, and historical events related events related to transgender individuals, role-play related to using transgender-affirming language and strengths-based reframing, demonstration for managing a client dyad Duration No concrete data available (> 110 min) Requirement status Voluntarily	19 undergraduate Bachelor of Social Work students	Students were evaluated by five licensed social workers who rated the performance by using the OSCE for Social Work Practice Performance Rating Scale. Each rater scored performance on 10 items according to a five-category rubric. Four subscales represent how students develop and use a collaborative relationship, conduct an ecosystemic assessment, set the stage for collaborative goal setting, and demonstrate cultural competence. The range of scores is from 10 to 50. Summing responses calculate the scores, and higher scores indicate more competence	19	Total scores on the Performance Rating Scale ranged from 22 to 40 with a mean of 31.6 out of 50 possible points. Students in the transgender simulation scenario scored lower on all four subscales compared to a similar cohort in a similar simulation experience with bisexual youth and cisgender parent dyads Students’ mean scores on the bisexual simulation experience were significantly higher than scores on the transgender simulation
Garcia- Acosta [60] (2019) Spain	Teaching method Multimodal Content Lectures with different experts (transgender people and families, experts in medicine, nursing, education, and law, anthropology and psychology) Film-forum group worked on the past and current history of transgender people by screening two films (The Danish Girl and About Ray) PBL group worked on parenthood in transgender people and aspects of this in assisted reproduction, as well as the current state of the situation Duration > 16 h in total according to time table, divided on three occasions Requirement status Voluntarily	116 nursing students (59 fourth year students in intervention groups G1 = 31 and G2 = 28 and another 57 third year students in the control group)	Knowledge Questionnaire about Transgender (KQaT, 30 items) was used to assess the previous knowledge level, questions were type test, the score from 0 to 10. The questionnaire was divided into four factors: biological, psychological, social, and legal Satisfaction questionnaire was completed by the intervention groups. A Likert scale was used (1 = strongly disagree to 10 = strongly agree) to respond to 25 items	*n* = 59 for intervention groups *n* = 57 for control group	After the intervention there were statistically significant differences in the level of knowledge of the fourth-year students compared to the third-year students (*p* = 0.000) No significance could be shown (*p* = 1.000) between film-forum and PBL. Both methodologies increased the level of knowledge Mean satisfaction score for film-forum was 8.04 out of 10 and for PBL it was 8.45 out of 10. There is no statistical significance between these figures

### Teaching format

#### Lectures and seminars

Nine studies used lectures (*N* = 6) or different kinds of seminars (*N* = 3) to educate about care of transgender individuals. Lecture audience consisted of medical (*N* = 4), pharmacy (*N* = 1) or physician assistant students (*N* = 1), and duration was 1 h (*N* = 3) or 2 h (*N* = 2). One study did not report time data.

Three studies chose a seminar format. Congdon et al. [61] presented a 45 min interactive session to medical and pharmacy students as well as health care professionals. Klotzbaugh et al. [62] worked with different teaching methods like stand-alone in-class, webinars, and online lectures and to integrate the subject into a three-semester curriculum of pre-licensure accelerated Bachelor nurses. Sherman et al. [63] gave a 90-min module for advanced practice nursing students including a presentation, discussion of case studies and open dialogue. Only two studies [58, 64] pointed out their lecturers had expertise in working with transgender clients, and one project included a member of the transgender community in the lecture team [65]. The remaining papers did not specify the proficiency of instructors.

### Standardised patient simulation activities

Seven studies designed a standardized patient simulation scenario around a transgender client with various settings and challenges but with the same central idea: to practice culturally sensitive communication and improve students’ confidence. Students were from the following professions: nursing (*N* = 4), social work (*N* = 1), medicine (*N* = 1), and one project was an IPE involving multiple professions.

The patient was portrayed by staff or people who received a training before the simulation (*N* = 3), real transgender identifying persons (*N* = 3), and one study worked with a high-fidelity manikin. Simulation experience took between 15- and 30 min excluding preparation time and debriefing. Three studies did not report time data. In the pilot study of Muckler et al. [66] nursing students conducted an anaesthesia preoperative interview in pairs with a MtF client with nausea and groin pain which turned out as testicular torsion. Montes-Galdeano et al. [52] created a scenario for nursing students around a patient who was admitted for bilateral mastectomy and got angry and weary when this person was misplaced in relation to gender or name. Additionally, a fellow nurse behaved in a transphobic way during handover.

Three simulations were set in an emergency setting. Maruca et al. [67] presented a MtF patient in early stages of transitioning who had extreme nervousness and migraine. The task for nursing students was to manage anxiety and maintain a safe environment during examination. Another study [59] involved 48-year-old MtF person with breast cancer where nursing students had to handle the clients’ hypercalcemia. Furthermore, Mc Cave et al. [53] developed an IPE activity around a FtM patient after a workplace assault, and participants completed a discharge meeting in multidisciplinary teams. Tyler et al. [56] evolved a dyadic simulation for social work students. The scenario took place at a family counselling centre where a young transgender client and cisgender parent made an appointment after the child revealed identifying as transgender.

The scenario created by Stumbar et al. [68] involved a FtM patient seeking care for abdominal pain. Several medical students had the opportunity to perform an inclusive sexual history.

### Multimodal approaches

Five studies combined several teaching methods in their intervention. Pechak et al. [54] created a 3-h IPE event including a presentation on interprofessional practice and transgender health. Additionally, students discussed the case of a transwoman with stroke in interprofessional and intraprofessional groups. The training for medical students by Thompson et al. [69] included a didactic portion with videos and lecture sl﻿ides, and a 3-h case based workshop with case discussions and role-play around a non-binary individual. On a separate day, they attended a 2-h panel discussion with members the transgender community and healthcare providers.

Allison et al. [55] implemented a 2-h IPE workshop involving presentations, videos produced by the  trans and non-binary community, group discussions and a patient panel. For nursing students, Garcia-Acosta et al. [60] designed a course containing a discussion forum, and two different group work activities with three sessions over three weeks. Participants were randomly assigned to one of the group interventions called PBL (problem-based-learning) or film-forum. Both intervention groups participated in discussions with experts of various professions, but worked separately according to their methodological strategy afterwards either on screening two films or dealing with a case about parenthood and assisted reproduction for transgender people.

Berenson et al. [70] offered a 2,5-h interactive module to medical students which contained a presentation on best practices of  transgender health care, small group discussions of videos about competent and poor ways of client-provider communication, and a patient panel with members of the trans community.

### Longitudinal projects

Besides training interventions included and evaluated in the review, three authors and their schools made the effort of repetitive content about transgender care within a longitudinal curriculum. The evaluation of outcomes of these additional educational events was not part of the individual report. Berenson et al. [70] described that in addition to the interactive module for second year medical students, Rutgers New Jersey Medical School included a first-year introductory module on LGBT health and clinical testing on LGBT.

The case-based workshop for second year medical students by Thompson et al. [69] aimed to expand the gender-affirming healthcare curriculum which already involves a 2-h transgender patient panel in the first year.

In addition to the 2,5-h workshop provided by Stumbar et al. [68] for second year medical students at Florida International University Herbert Wertheim College of Medicine, they have opportunity to attend a LGBTQ patient panel in their second year and a 2-h course on sexuality theory and LGBTQ health disparities in first year.

### Interprofessional education activities

Three interventions were planned as IPE activities to generate interprofessional cooperation and communication about topics which affect many health care related professions. Pechak et al. [54] included students of occupational therapy, pharmacy, physical therapy, rehabilitation counselling, social work, speech language pathology and prospectively nursing students and developed a case of transwoman with a stroke to discuss in intra-and interprofessional teams about barriers and challenges the patient faced during transition.

Allison et al. [55] let students of health professions and nursing discuss about videos produced by the trans and nonbinary community. The clips showed positive and negative patient provider interactions in different settings. McCave et al. [53] facilitated collaboration between medical, nursing, occupational therapy, physical therapy, Physician assistant, social work, and health care administration program students by establishing a standardized patient case. A FtM identifying person had an ankle injury, and students had to complete a team huddle followed by a discharge planning meeting with the patient. Earlier participants watched a video about the initial interaction with the patient and discussed the behaviour of providers.

### Outcome measures: Quantitative findings

#### Knowledge

Fourteen studies analysed the impact of a training intervention on knowledge about various topics like terminology, health disparities, medical interventions, health policy, community resources, examination and sexual history and cultural competence. Knowledge changes were evaluated by using non-standardised and self-developed surveys [55, 58, 60, 61, 64, 68, 71], adapted a pre-existing measure [57, 62, 63, 65, 66, 69] or previously validated tools [59]. The quantity of knowledge assessment items was highly variable.

Nine studies assessed knowledge gains by comparing right answers between pre-and postinterventional surveys [55, 57, 60–62, 65, 68] or between different groups [58, 71]. Remaining projects (*N* = 5) employed self-assessment questions to rate knowledge in general or on special transgender related issues [59, 63, 64, 66, 69].

Almost all programs (*N* = 13) identified a statistically significant change in knowledge scale total scores [55, 58–62, 69] or single items [57, 63–65, 68, 71] after the intervention. One study [66] only compared numerical data on yes/no questions without doing a statistical analysis. Seven out of 28 students answered to have adequate knowledge to address trans health care needs before the intervention. After the simulation activity nine students felt sufficiently knowledgeable.

All three projects [58–60] which used a study design with an intervention and control group found statistically significant higher scores for the intervention group compared to the control after the training intervention. In addition, one of these studies compared the results between two different intervention groups which worked according to different methodological strategies, but no significant differences were shown [60]. Another study [71] analysed knowledge differences between medical students from first to fourth year after receiving one or two lectures. Individual knowledge performance did not significantly differ by year of training with the exception of two items. Only one study [57] measured knowledge retention 1 year after students attended a 1-h lecture and indicated a consistent high rate of correct or favourable responses and a significant increase concerning one particular item.

#### Attitude

Ten educational interventions aimed to promote more favourable attitudes towards transgender individuals. Changes were assessed using validated scales [55, 59, 62, 67], adapted or extended versions of published tools [57, 63, 65, 66, 69] or survey questions exclusively developed for the project [64]. Results appeared inhomogeneous with seven studies recording significant changes and three projects which did not. Significant improvements were shown in mean score [62] or total scores [59, 67], subscales [55] or single questions [57, 65], and one study reported a significant decrease concerning one item [63]. This item covered the importance for nursing students to know about the patient`s gender identity. Authors explanations for the decrease were changes to sample composition or students` understanding that knowing details about an individual´s gender identity is not obligatory to provide respectful care.

One study which deployed a control group detected significant differences between intervention and control group scores within an affective scale [59]. Long-term effects of a training intervention on attitudes were analysed by only one study after 1 year [57]. Authors reported consistently positive attitudes and even observed a significant increase regarding students ‘comfort with a clinical scenario involving a transgender person. Indicating more comfort with transgender clients was rated as having favourable attitudes. Two studies could not determine significant changes in participants’ attitudes after the education [64, 69], and one study did not perform a statistical analysis but counted the answers [66] to yes/no questions.

### Confidence and comfort

Nine studies explored the effect of education about transgender care on students ‘comfort or confidence level, where the authors addressed the following aspects: comfort with providing gender-affirmative care or interacting with transgender clients [54, 57, 58, 61, 65, 66, 68, 70, 72] and comfort with hormone therapy [70, 72] or unique health risks [70]. Assessment tools used 5-point Likert scale [54, 58, 61, 65, 68, 70, 72], four level scale [57] or a yes/no question format [66].

Six studies found significant improvements in confidence for providing competent care within a pre-post comparison [57, 61, 65, 68, 70, 72]. Teaching formats were lectures [57, 61, 65, 72], standardised patient scenarios [68] or multimodal workshops [70]. But only the study by Najor et al. [57] provided long-term data on comfort levels after 1 year. A follow-up survey identified continuing high comfort levels with treating transgender individuals, and with a clinical scenario involving discordance of gender expression and sex listed in health records. Authors interpreted the fact of being more comfortable as having more favourable attitudes towards transgender clients. Thus, clear differentiation between attitude and comfort was not feasible.

Three studies [54, 58, 66] did not perform statistical analyses comparing students’ answers before and after the training intervention. Two of them [54, 58] only had a postsurvey design and one study counted answers to yes/no items [66]. Instead of this approach Ostroff et al. [58] compared pharmacy students who attended a lecture and those who did not regarding comfort levels with providing care and prescription counselling to a transgender client. No significant difference in means was detected. Pechak et al. [54] asked if students were comfortable interacting with a transgender person and comfortable with providing services. A significant difference between these items was found, implying higher confidence for providing services compared to personal interactions.

### Practical skills

Five studies addressed the development of practical skills when interacting with transgender clients due to participating in a special learning activity. Four programs [53, 59, 64, 69] used self-assessment questionnaires, and one study applied an external assessment [56]. Skills were assessed with pre-existing tools [56, 59], adapted assessments [53, 64, 69] or a survey exclusively designed for the intervention [64]. Three studies reported significant changes between pre and postintervention skills scores [59, 64, 69]. In addition, Ozkara San [59] compared results between an experimental group and a control group but did not detect significant differences.

Tyler et al. [56] designed an intervention in which the performance of students in a simulation scenario around a transgender child and parent was rated by five licensed social workers. Mean performance scores were compared to a similar student cohort which participated in simulation around a bisexual youth and a cisgender parent. Results showed a statistically worse performance on the scenario involving a transgender person.

McCave et al. [53] created an IPE simulation activity with a transgender client. Students reported a high preparation level for interprofessional practice in a postsurvey, but not specifically their skills to provide care for transgender individuals. Averaging the data of 2 years revealed that at least 90% felt better prepared working on an interprofessional team to discharge a transgender patient by answering “strongly agree” or “agree” on each core competence.

### Satisfaction and effectiveness of intervention

Eight studies analysed the effectiveness of their intervention and participants’ satisfaction. An evaluation was performed for one seminar-type program [61], four simulation-based activities [53, 59, 66, 68] and three multimodal workshops [54, 60, 70]. Congdon et al.[61] reported that 90% of participants agreed or strongly agreed the seminar was applicable to their clinical setting, and moreover increased their confidence levels to provide care to transgender clients which was acknowledged by 84%. Looking at simulation experiences Muckler et al. [66] pointed out students verbally confirmed the simulation was helpful, effective and increased their ease of caring for trans patients. This is in accordance with another study [68] in which above 90% of students felt better equipped in knowledge and skills after the simulation. In addition, the majority found the case believable (93%) and the debrief helpful (93%).

Ozkara San [59] reported high mean scores for the evaluation of pre-briefing, simulation and debriefing indicating great satisfaction with the components. Furthermore, students assessed on a 10-point scale if the patient scenario helped to develop knowledge, cultural assessment and interview skills, culturally sensitive attitudes and their overall confidence. A significant positive correlation was found between these four questions and experimental groups´ post-test Transcultural self-efficacy tool (TSET) responses.

In the workshop of McCave et al. [53] a large majority of students identified the panel presentation of a local community organization promoting awareness and understanding for the LGBT community as the most valuable component, followed by the discharge planning meeting with the standardized patient. Nearly similar results were delivered by Berenson et al. [70] where participants’ mean ratings were highest for the patient panel, followed by the large-group didactic lecture, and the small-group video session. Additionally, 58 students highlighted the value of the panel in free-text answers.

Garcia-Acosta et al. [60] compared students’ satisfaction scores of two different teaching methodologies called PBL and film-forum, but did not find a statistically significant difference. Another study [54] assessed the impact of a multimodal workshop on students´ preparedness for interprofessional practice by using the Readiness for interprofessional learning scale (RIPLS) and Interprofessional attitudes scale (IPAS). A significant change for both surveys demonstrated that knowledge and attitude related to interprofessional collaboration improved.

### Outcome measures: Qualitative findings

Montes-Galdeano et al. [52] performed a descriptive qualitative study with first-year nursing students. Data was collected after participation in a clinical simulation scenario. Students performed a nursing assessment with a transgender patient and did a handover to a fellow nurse who showed transphobic and discriminatory attitudes afterwards. 12 focus groups’ interviews between 40 and 60 min were conducted by using a semi-structured guide.

Most students thought the simulation was effective to gain better understanding and awareness of transgender patients’ needs and increased the perceived importance to achieve a positive impact on care. They noticed knowledge gaps on personal and professional levels, and a lack of experience in communication with transgender individuals during a nursing assessment or when talking about the patient during a handover. Proper knowledge about terminology, transition process or hormonal medication was considered as important to promote care. Therefore, the need of integrating LGBTQ + content into a curriculum was stated. Almost all students identified the fellow nurse`s discriminatory and transphobic comments, but her behaviour was rarely criticised or corrected. Many students also mentioned the need for structural-level changes and identified that senior charge nurses and other health administrators play an essential role to overcome inequalities, and to support their professionals’ knowledge and positive attitudes towards the transgender community.

### Quality assessment and risk of bias within the studies

The critical appraisal of included studies was done by the first author. All quality ratings are shown in Supplementary materials 4b-c. As mentioned above we applied different tools for evaluating quantitative and qualitative research. The qualitative study by Montes Galdeano et al. [52] scored 17 out of 20 possible points, indicating overall good quality. Areas of weakness were the convenience sample, the lack of reflexivity about the authors` possible influences on research, and the data analysis process. To assess the quality of 20 quantitative studies we used an 18-item checklist and counted the number of questions evaluated with a “yes”. The highest count was 17 items, and the lowest rating was 8. The appraisal of the remaining studies was between 9 and 13 “yes” items.

The most common shortcomings were identified by analysing which items got little “yes” scores. Weak points included the following: lack of control for confounding factors, no report of estimates of variance, use of non-valid outcome measures, insufficient description of participant characteristics and low response rates below 80% among participants to surveys and tools. Only one study [60] considered the potential for confounding factors and performed a homogeneity test between their 3 groups and an ANCOVA to eliminate the students’ prior knowledge as a covariate which may have influenced the degree of subsequent knowledge assessments. The remaining studies have a potential risk for confounders which could have influenced study results. For example, participants’ answers in surveys may be affected by social desirability as well as other curriculum activities, and self-study may could have had an impact on outcomes, in particular when there was a large interval between postsurvey and intervention.

Moreover, the description of participant characteristics often was incomplete since demographics and some students’ characteristics were missing, or a selection strategy was not clearly described. In many cases participation was voluntary, and which can be subject to selection bias. These circumstances and the point that most studies represented just one site limit the representativeness of results.

Regarding outcome measures used only three studies applied valid and reliable tools. Another ten reports were valued with “partial” because at least the reliability of assessment tools was tested. A further important fact is that only one study [60] implemented a randomization of students into two different intervention groups, and therefore got a “yes” for Item 6 in our checklist. The control group included students of another year of study. For all other studies the item was not applicable because either no second group as a control was established, or the control group included students of another course or year and no randomization was conducted. Less frequently observed shortcomings were missing information about intervention characteristics such as time data, a small sample size or insufficiently detailed reports of results. Data analysis and statistical tests were mostly justified and appropriate.

## Discussion

Through our systematic review we aimed to illustrate the current state of training interventions to educate health or allied health profession students about transgender individuals and their unique challenges, in particular in health care settings, and to observe the influence of education on clients’ care.

Investigating this subject is vitally important because transgender people often face discrimination, stigmatization, victimisation, and violence, and which are common risk factors impairing mental health [73, 74]. Thus, a high proportion of transgender clients reports to suffer from depression, anxiety, self-harm, or attempted suicide [13, 75–77]. Studies stated that transgender individuals more frequently rate their overall health as fair or poor compared to cisgender individuals, and have higher health service use, in particular  for mental health and self-harm [76, 77]. But it is also known that health care providers and students lack in knowledge and training to provide competent care [78–80].

We found a multitude of educational interventions across numerous health or allied health professions but mainly for medical or nursing students. However, we noticed education approaches of other professions such as pharmacy, social work, or physician assistants. The subject of culturally competent and gender-affirmative care concerns a wide range of disciplines, and evidence proves a lack of preparedness and knowledge about unique needs and problems of transgender clients across many professions [80–85]. Therefore, a positive aspect to highlight is the effort of three authors to design an IPE activity by using case discussion in groups [54], video discussions [55] or standardised patient simulations [53]. Two of these studies [53, 54] supported the promising results of several reviews [86–89] that interprofessional education appeared to increase effective communication, trains teamwork skills and collaboration with other health care professions and improves attitudes regarding interprofessional interaction. The remaining study [55] did not analyse outcomes in terms of IPE competencies.

All included studies differ widely regarding teaching time and format, number and profession of participants and delivered content. Commonly discussed topics were terminology, aspects of gender affirmation, health inequities and culturally sensitive communication﻿, whereas the needs of transgender youth and non-binary or other gender diverse individuals were underrepresented.

Our second target was to explore if training interventions for future health professionals can positively influence the care for transgender clients. Results to this question were ambiguous. Almost all interventions (*N* = 19) pointed a favourable effect on some or multiple domains of students’ knowledge, attitude, confidence and comfort levels or practical skills regarding care for transgender individuals. Most of them showed significant improvements either in mean or total scores, subscales, or single items regardless of which teaching method was used. Exceptions were two reports because of their study design: the descriptive qualitative study by Montes-Galdeano et al. [52] and the intervention of Tyler et al. [56] in which students’ performance skills in a scenario around a transgender client and parent were compared with another group of students who participated in a bisexual/parent scenario. Nevertheless, the overall trend is promising because higher knowledge scores seem to be associated with more favourable beliefs about LGBT health care and gender identity, open communication behaviours and larger perceived importance of sensitivity and communication skills training [90].

But there is a clear lack of long-term data to rate effectiveness of training interventions in the long run. Only one study [57] measured knowledge retention and attitudes 1 year after lecture delivery and showed promising high rates of correct or favourable responses. Another study by Allison et al. [55] aimed to assess the long term impact after nine months, but the response rate was too low to perform an analysis. A further point to discuss is if the observed positive post intervention changes will lead to improved outcomes for transgender clients, patient-provider relationships, and culturally sensitive and gender-affirmative care. Furthermore, it remains unknown if a one-day activity is sufficient to gain competence or if longitudinal curricula and continuous training are needed. Three authors of included studies [68–70] reported their health profession schools integrated more education on transgender or LGBTQ health into their curricula besides the presented activity.

Due to the heterogeneity of study designs, assessment tools, and outcome measures we did not perform a meta-analysis and cannot state which teaching format was most effective and may reflect best practice. There is also a lack of comparative studies to compare different teaching formats. One included study by Garcia Acosta et al. [60] had two intervention groups in which students worked according to different methodologies. Knowledge outcomes of both groups were significantly improved after training but did not differ significantly.

We are aware that the extent and design of an educational intervention depends on several factors such as time frame in a curriculum, available locations and commitment of teaching staff, experts, and the transgender community. The lack of curriculum time can be an explanation for lectures or interactive seminars being the most common teaching format in this review. That would be in line with study results showing that a lack of time was pointed out to be an important barrier to include the topic of transgender care [91, 92]. The subject is often taught within a broader complex about LGBTQI care [91, 93, 94] making it difficult to disentangle how much time was spent exclusively on care for transgender clients. In addition, the number of hours dedicated to LGBTQI health is highly variable [81, 92, 94] but has a major influence on students’ preparedness, attitudes, and knowledge along with the exposure to LGBT clients [81]. But as several studies identified providers have lower knowledge and feel more discomfort when caring for transgender individuals compared to LGB people [84, 95–97] it may be wise to discuss the subject separately. This is in line with the results of Tyler et al. [56] who described their students performed significantly worse in a scenario with a transgender client than with a bisexual client.

An international survey from 2012 [8] with 93,079 LGBTQ respondents from EU member states indicated that trans individuals more frequently felt personally discriminated against or harassed in different settings when compared to LGB groups. Moreover, more often they were victims of violence more often. The survey update from 2019 [10] again confirmed the highest rates of LGBTI-related harassment among trans- and intersex respondents.

Another identified barrier for teaching about transgender care is the lack of expertise among faculty [92], so it may be meaningful to involve the transgender community in planning process and implementation of a workshop. Nine studies in our review (42.9%) have successfully collaborated with transgender or non-binary individuals as lecturers, standardised patients, or participants in panel discussions or videos [52, 53, 55, 59, 60, 65, 68–70]. In particular the patient panel with members of the community was seen as the most valuable of two different projects [53, 70], and standardised patient scenarios with transgender clients increased students’ knowledge, skills [59, 68] or raised awareness about patient’s needs [52]. Although seven projects contained a standardised patient experience only three of them had a transgender person portraying the patient. Two authors explained they would prefer transgender individuals playing the patient because they certainly can give the most accurate and authentic representation, but the recruitment process was challenging [53, 68].

Working together with transgender individuals provides an opportunity to gain an insight to challenges they face in health care settings and talk about their main concerns and recommendations how care can be improved. When asking transgender people about health care several studies indicated a call for increased education and training for health professionals [26, 98, 99] to achieve competence. There is no uniform definition of competence on this matter, and which attributes and abilities are needed. In the broadest sense it is composed of knowledge, skills and other components such as attitude and values [100], and enables the person to do something well.^1^ In terms of gender-affirmative care this includes knowledge of best practice, use of correct name and pronouns, and refrain from asking inappropriate and irrelevant questions about their transgender experience [26, 27, 99, 101]. Several individuals reported feeling anxious when visiting new healthcare providers because of not knowing their knowledge and competency level [101], or already expect the health professionals to have a knowledge gap. Due to this perceived lack of knowledge some individuals had to educate the healthcare personnel during their encounters [27].

### Implications for future research and policy

The present findings of this review suggest that training interventions have a favourable impact on students’ competence development regarding transgender care. But it remains unknown if this effect is long-lasting since long-term data is lacking, and which is an area future research should address. As there is much diversity among teaching formats, content and time frame, future studies could consider the best practices to achieve set goals. Further context that can be discussed are the advantages of IPE activities compared to single profession teaching, or whether a one-day intervention sufficiently improves competence or continuing education is necessary. We identified three schools who integrated more transgender topics in a longitudinal curriculum, and a comparison﻿ with one-day projects regarding the outcomes of interest may be interesting.

The most important area to explore is how education of future providers affects clinical outcomes for transgender clients, and if they actually experience improved and competent care or acknowledge positive changes. We believe the dialogue between professionals or future health care providers and the transgender community is of particular importance, and therefore recommend the cooperation with community organizations and participation of trans identifying individuals in such training interventions.

During literature research we recognized that there are significantly fewer projects exclusively on care of transgender individuals than about LGBTQ or sexual and gender minorities in general. But since many providers feel more incompetent when caring for transgender people than LGB individuals it seems meaningful to plan education about transgender care separately.

Our implications for policy development could potentially be the funding of future research and training interventions on transgender care, as well as performing nationwide surveys to gain insight in the challenges and life reality of transgender individuals to derive necessary actions and eliminate disparities.

### Limitations

One major shortcoming of this review is the lack of long-term data which was provided by only one study after 1 year. Furthermore, there are limitations due to search strategy and inclusion criteria, and hence this review does not reflect the totality of current training interventions about this particular topic between 2017 and 15 June 2021. First: it is subject to publication bias because only the listed databases were searched, and grey literature was excluded. Second: publication language was defined as English or German. Third: the study population had to contain students of health or allied health professions. Therefore, we excluded interventions designed for professionals or residents. Additionally, the largest part of studies was conducted in the U.S.A, so transferability of results is limited, and the presentation of outcomes is biased.

Another factor to consider is the lack of well-conducted studies due to the absence of validated assessment tools or control groups and tests for confounding. Moreover, a risk of selection bias exists owing to voluntary participation in the training intervention or voluntary response to questionnaires. Students may already have greater interest and more favourable attitudes before the intervention.

Besides, generalization of study results is restricted because of the design as single-institution projects, the lack of descriptions of students’ characteristics and demographics, and low response rates. Future studies may pay attention to good methodological quality in order to supply more reliable results.

## Conclusion

The lack of knowledge and skills of providers to effectively meet the (at least partially self-percieved) needs of transgender clients could be one source of health inequities and negative experiences. Therefore, it is imperative to include content about this population into curricula of health and allied health professions. This systematic review highlights the positive effects of a variety of educational interventions on students’ knowledge, skills, attitudes, and confidence levels. However, improvements were only measured in the short-term, and future research should focus on collecting long time data. Furthermore, the use of control groups and standardised and valid outcome assessments is desperately needed to compare numerous different interventions, and to determine what is best practice. Besides it still remains unknown if those concerned—people identifying as transgender—experience noticeable improvements in health care, and which is another area to explore in future research.

### Supplementary Information

Below is the link to the electronic supplementary material.

Supplementary file1 (PDF 314 KB)

## Data Availability

This publication covers a systematic literature review and no original data. Because of this, no original data can be made available.

## Footnotes

1 Definition of Oxford Advanced Learner's Dictionary.
