# Supplementary material for: Delivering transgender-specific knowledge and skills into health and allied health studies and training: a systematic review
Source: Eur Child Adolesc Psychiatry. 2023 Apr 28;33(5):1327–54. doi: 10.1007/s00787-023-02195-8 (PMC11098887; doi:10.1007/s00787-023-02195-8)
Supplement: Supplementary file 1 — Supplementary file1 (PDF 314 KB) [file 787_2023_2195_MOESM1_ESM.pdf]

SUPPLEMENTARY MATERIALS

1. List of abbreviations

ANCOVA	Analysis of Covariance
DSM-5	Diagnostic and Statistical Manual of Mental Disorders V5
EU	European Union
FtM	Female-to-Male
ICD	International Classification of Diseases and Related Health Problems
IPE	Interprofessional Education
LGBTQI	Lesbian, Gay, Bisexual, Trans, Queer and Intersex
MtF	Male-to-Female
OSCE	Objective structured clinical examination
PBL	problem-based learning
U.S.A.	United States of America

2. Literature Research of Databases

a. Detailed Flowchart of study selection process

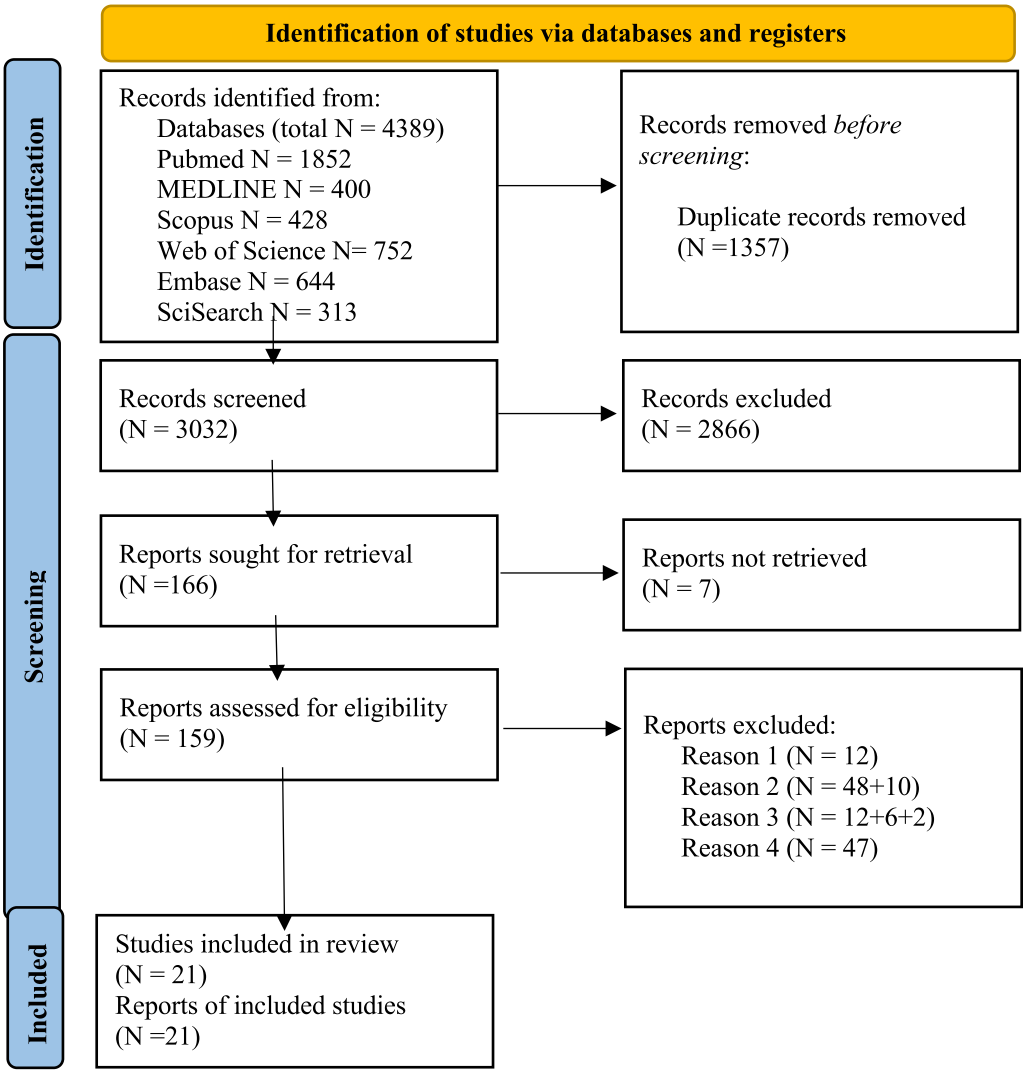

From: Page MJ, McKenzie JE, Bossuyt PM, Boutron I, Hoffmann TC, Mulrow CD, et al. The PRISMA 2020 statement: an updated guideline for reporting systematic reviews. BMJ 2021;372:n71. Doi: 10.1136/bmj.n71

Reason 1: Report was already used in a prior review

Reason 2: Study contained no transgender-specific content (LGBTQI in general) or no content about transgender care at all

Reason 3: Publication type: review, conference abstract, commentary

Reason 4: Study design: no training, no students of health or allied health professions,

b. Detailed search strategy for each database

1. Pubmed

Data access: 22 May 2021

Search: **((Gender and (dysphoria or incongruence or identity or disorder or minority)) or (transgender or transsexual or transexual or transfemale or transwoman or transwomen or transman or Transmen or transpeople)) and (training or Teaching or curriculum) and student and health** Filters: **from 2017 – 2021**

(((((“gender identity”[MeSH Terms] OR (“gender”[All Fields] AND “identity”[All Fields]) OR “gender identity”[All Fields] OR “gendered”[All Fields] OR “gender s”[All Fields] OR “gendering”[All Fields] OR “genderized”[All Fields] OR “genders”[All Fields] OR “sex”[MeSH Terms] OR “sex”[All Fields] OR “gender”[All Fields]) AND (“dysphoria”[All Fields] OR “dysphorias”[All Fields] OR (“incongruence”[All Fields] OR “incongruences”[All Fields] OR “incongruencies”[All Fields] OR “incongruency”[All Fields] OR “incongruent”[All Fields] OR “incongruently”[All Fields] OR “incongruities”[All Fields] OR “incongruity”[All Fields] OR “incongruous”[All Fields] OR “incongruously”[All Fields]) OR (“identities”[All Fields] OR “identity”[All Fields]) OR (“disease”[MeSH Terms] OR “disease”[All Fields] OR “disorder”[All Fields] OR “disorders”[All Fields] OR “disorder s"[All F”elds] OR "disord“s"[All F”elds]) OR ("mino“ity group”"[meSH Terms] OR“(“minority”[All Fields] AND “groups”[All Fields]) OR “minority groups”[All Fields] OR “minorities”[All Fields] OR “minority”[All Fields] OR “minority s”[All Fields] OR “minors”[MeSH Terms] OR “minors”[All Fields] OR “minor”[All Fields]))) OR (“transgender persons”[MeSH Terms] OR (“transgender”[All Fields] AND “persons”[All Fields]) OR “transgender persons”[All Fields] OR “transgender”[All Fields] OR “transgendered”[All Fields] OR “transgenders”[All Fields] OR (“transsexual”[All Fields] OR “transsexual s”[All Fields] OR “transsexualism”[MeSH Terms] OR “transsexualism”[All Fields] OR “transsexuality”[All Fields] OR “transsexuals”[All Fields]) OR (“3ranssexuality”[All Fields] OR “transgender persons”[MeSH Terms] OR (“transgender”[All Fields] AND “persons”[All Fields]) OR “transgender persons”[All Fields] OR “transexual”[All Fields] OR “transexuals”[All Fields]) OR “transfemale”[All Fields] OR “transwoman”[All Fields] OR “transwomen”[All Fields] OR “transman”[All Fields] OR “Transmen”[All Fields] OR “transpeople”[All Fields])) AND (“education”[MeSH Subheading] OR “education”[All Fields] OR “training”[All Fields] OR “education”[MeSH Terms] OR “train”[All Fields] OR “train s”[All Fields] OR “trained”[All Fields] OR “training s"[All Fields] “R "traini”gs"[All Fields] “R "train”"[all fields] OR“("educati”n"[MeSH Subheading] OR “education”[All Fields] OR “teaching”[All Fields] OR “teaching”[MeSH Terms] OR “teaches”[All Fields] OR “teach”[All Fields] OR “teachings”[All Fields] OR “teaching s”[All Fields]) OR (“curriculum”[MeSH Terms] OR “curriculum”[All Fields] OR “curricula”[All Fields] OR “curriculums”[All Fields] OR “curriculum s”[All Fields] OR “education”[MeSH Subheading] OR “education”[All Fields]))) AND ((“student s”[All Fields] OR “students”[MeSH Terms] OR “students”[All Fields] OR “student”[All Fields] OR “students s”[All Fields]) AND (“health”[MeSH Terms] OR “health”[All Fields] OR “health s”[All Fields] OR “healthful”[All Fields] OR “healthfulness”[All Fields] OR “healths”[All Fields]))) AND (2017:2021[pdat])

2. Medline

Data access: 15.06.2021

Search: (((Gender* and (dysphori* or incongruen* or identit* or disorder* or minorit*)) or (transgend* or transsex* or transex* or transfem* or transwom* or transma* or Transmen* or transperson* or transpeopl*) and (train* or Teach* or curricul*) and student* and health*

filter: All fields

Platform: Ovid MEDLINE(R) and In-Process, In-Data-Review & Other Non-Indexed Citations 1946 to December 08, 2021

3. Scopus

Data access: 09.06.2021

Search: (((Gender* and (dysphori* or incongruen* or identit* or disorder* or minorit*)) or (transgend* or transsex* or transex* or transfem* or transwom* or transma* or Transmen* or transperson* or transpeopl*) and (train* or Teach* or curricul*) and student* and health*

filter :Abstract and title

4. Web of Science

Data access: 15.06.2021

(((Gender* and (dysphori* or incongruen* or identit* or disorder* or minorit*)) or (transgend* or transsex* or transex* or transfem* or transwom* or transma* or Transmen* or transperson* or transpeopl*) and (train* or Teach* or curricul*) and student* and health*

filter: Topic

5. Embase

Data access: 17.06.2021

Search: (((Gender and (dysphoria or incongruence or identity or disorder or minority)) or (transgender or transsexual or transexual or transfemale or transwoman or transwomen or transman or Transmen or transpeople)) and (training or Teaching or curriculum) and student and health

Platform: STN International

3. Quality assessment of studies

a. 18-item self-developed assessment tool

**Criteria**	**Yes**	**Partial**	**No**	**N/A**
1.Was the study question/aim/objective clearly stated?				
2.Were eligibility/selection criteria for the study population prespecified and clearly described?				
3. Are the subject characteristics sufficiently described?				
4.Were the participants in the study representative of those who would be eligible for the intervention in the population of interest?				
5.Was the sample size sufficiently large to provide confidence in the findings?				
6.If random allocation to treatment group was possible, is it described?				
7.Was the intervention clearly described and delivered consistently across the study population?				
8.Were the main outcome measures/data collection tools used valid and reliable?				
9.Were the data analysis methods used to assess the main outcomes appropriate?				
10. Did the data analysis examine changes in outcome measures from before to after the intervention?				
11. Were statistical tests done that provided P values for the pre- post changes or intergroup differences?				
12.Are the results reported in sufficient detail?				
13. Was there some estimate of variance reported for the main outcomes?				
14-Were study results controlled for confounding?				
15.Were the postsurvey rate or loss to follow up after baseline 20% or less?				
16.Is the conclusion supported by the results?				
17.Were study limitations or potential bias discussed?				
18. Did the authors declared conflicts of interest?				

b. Quality assessment of quantitative studies

Y = yes

P = partial

N = no

N/A = not applicable

First Author	Hart [64]	Stumbar [68]	Ostroff [58]	Sherman [63]	Turban [71]	Thompson [69]	Congdon [61]	Mc Cave [53]
Criteria								
1.Was the study question/aim/objective clearly stated?	Y	Y	Y	Y	Y	Y	Y	Y
2.Were eligibility/selection criteria for the study population prespecified and clearly described?	P	Y	Y	Y	Y	Y	P	P
3. Are the subject characteristics sufficiently described?	Y	N	N	Y	N	Y	N	N
4.Were the participants in the study representative of those who would be eligible for the intervention in the population of interest?	P	P	P	Y	Y	Y	P	P
5.Was the sample size sufficiently large to provide confidence in the findings?	Y	Y	Y	Y	Y	Y	Y	Y
6.If random allocation to treatment group was possible, is it described?	N/A	N/A	N/A	N/A	N/A	N/A	N/A	N/A
7.Was the intervention clearly described and delivered consistently across the study population?	Y	Y	Y	Y	P	Y	P	Y
8.Were the main outcome measures/data collection tools used valid and reliable?	P	N	N	N	N	P	P	P
9.Were the data analysis methods used to assess the main outcomes appropriate?	Y	Y	Y	Y	Y	Y	Y	Y
10. Did the data analysis examine changes in outcome measures from before to after the intervention?	Y	Y	N	Y	N	Y	Y	N
11. Were statistical tests done that provided P values for the pre- post changes or intergroup differences?	Y	Y	Y	Y	Y	Y	Y	N
12.Are the results reported in sufficient detail?	Y	Y	Y	Y	Y	P	P	Y

13. Was there some estimate of variance reported for the main outcomes?	N	N	N	N	N	N	N	N
14-Were study results controlled for confounding?	N	N	N	N	N	N	N	N
15.Were the postsurvey rate or loss to follow up after baseline 20% or less?	N	P	P	N	N	N	Y	N
16.Is the conclusion supported by the results?	Y	Y	Y	Y	Y	Y	Y	Y
17.Were study limitations or potential bias discussed?	Y	Y	Y	Y	Y	Y	Y	Y
18. Did the authors declared conflicts of interest?	Y	Y	Y	Y	Y	Y	Y	Y

**First Author**	**Najor [57]**	**Muckler [66]**	**Maruca [67]**	**Arora [65]**	**Klotzbaugh [62]**	**Pathoulas [72]**	**Ozkara San [59]**	**Pechak [54]**
Criteria								
1.Was the study question/aim/objective clearly stated?	Y	Y	Y	Y	Y	Y	Y	Y
2.Were eligibility/selection criteria for the study population prespecified and clearly described?	Y	P	P	P	P	P	P	P
3. Are the subject characteristics sufficiently described?	Y	N	Y	N	Y	N	Y	N
4.Were the participants in the study representative of those who would be eligible for the intervention in the population of interest?	Y	P	P	P	P	P	P	P
5.Was the sample size sufficiently large to provide confidence in the findings?	Y	P	Y	Y	P	Y	Y	Y
6.If random allocation to treatment group was possible, is it described?	N/A	N/A	N/A	N/A	N/A	N/A	N/A	N/A
7.Was the intervention clearly described and delivered consistently across the study population?	Y	P	P	Y	Y	Y	Y	Y
8.Were the main outcome measures/data collection tools used valid and reliable?	P	N	Y	P	P	N	Y	Y
9.Were the data analysis methods used to assess the main outcomes appropriate?	Y	Y	Y	Y	Y	Y	Y	Y
10. Did the data analysis examine changes in outcome measures from before to after the intervention?	Y	Y	Y	Y	Y	Y	Y	Y

11. Were statistical tests done that provided P values for the pre- post changes or intergroup differences?	Y	N	Y	Y	Y	Y	Y	Y
12.Are the results reported in sufficient detail?	Y	Y	Y	Y	Y	Y	Y	Y
13. Was there some estimate of variance reported for the main outcomes?	N	N	N	N	N	N	N	N
14-Were study results controlled for confounding?	N	N	N	N	N	N	Y	Y
15.Were the postsurvey rate or loss to follow up after baseline 20% or less?	N	Y	N	P	Y	Y	Y	Y
16.Is the conclusion supported by the results?	Y	Y	Y	Y	Y	Y	Y	Y
17.Were study limitations or potential bias discussed?	Y	Y	Y	Y	Y	Y	Y	Y
18. Did the authors declared conflicts of interest?	Y	Y	Y	Y	Y	Y	Y	Y

First Author	**Berenson [70]**	**Tyler [56]**	**Garcia Acosta [60]**	**Allison [55]**
Criteria				
1.Was the study question/aim/objective clearly stated?	Y	Y	Y	Y
2.Were eligibility/selection criteria for the study population prespecified and clearly described?	Y	P	Y	P
3. Are the subject characteristics sufficiently described?	N	N	Y	N
4.Were the participants in the study representative of those who would be eligible for the intervention in the population of interest?	P	P	Y	P
5.Was the sample size sufficiently large to provide confidence in the findings?	Y	P	Y	Y
6.If random allocation to treatment group was possible, is it described?	N/A	N/A	Y	N/A
7.Was the intervention clearly described and delivered consistently across the study population?	Y	Y	Y	Y
8.Were the main outcome measures/data collection tools used valid and reliable?	N	P	P	P
9.Were the data analysis methods used to assess the main outcomes appropriate?	Y	Y	Y	Y
10. Did the data analysis examine changes in outcome measures from before to after the intervention?	Y	Y	N	Y
11. Were statistical tests done that provided P values for the pre- post changes or intergroup differences?	Y	Y	Y	Y
12.Are the results reported in sufficient detail?	Y	Y	Y	Y
13. Was there some estimate of variance reported for the main outcomes?	N	Y	Y	Y
14-Were study results controlled for confounding?	N	N	Y	N
15.Were the postsurvey rate or loss to follow up after baseline 20% or less?	Y	N/A	Y	Y
16.Is the conclusion supported by the results?	Y	Y	Y	Y
17.Were study limitations or potential bias discussed?	Y	Y	Y	Y
18. Did the authors declared conflicts of interest?	Y	Y	Y	Y

c. Quality Assessment for qualitative study of Montes-Galdeano [52]

Criteria	Yes	Partial	No
1. Question/objective sufficiently described?	X		
2. Study design evident and appropriate	X		
3.Context for the study clear?	X		
4. Connection to a theoretical framework/wider body of knowledge?	X		
5. Sampling strategy described, relevant and justified?		X	
6. Data collection methods clearly described and systematic?	X		
7. Data analysis clearly described and systematic?	X		
8. Use of verification procedures to establish credibility?	X		
9.Conclusions supported by the results?	X		
10. Reflexivity of the account?			X
